# Disease-Specific Risk Models for Predicting Dementia: An Umbrella Review

**DOI:** 10.3390/life14111489

**Published:** 2024-11-15

**Authors:** Eugene Yee Hing Tang, Jacob Brain, Serena Sabatini, Eduwin Pakpahan, Louise Robinson, Maha Alshahrani, Aliya Naheed, Mario Siervo, Blossom Christa Maree Stephan

**Affiliations:** 1 Population Health Sciences Institute, Newcastle University, Newcastle NE2 4HH, UK; a.l.robinson@newcastle.ac.uk; 2 Institute of Mental Health, Mental Health and Clinical Neurosciences, School of Medicine, University of Nottingham, Nottingham NG7 2UH, UK; jacob.brain@nottingham.ac.uk (J.B.); blossom.stephan@curtin.edu.au (B.C.M.S.); 3 Freemasons Foundation Centre for Men’s Health, Discipline of Medicine, School of Psychology, The University of Adelaide, Adelaide, SA 5005, Australia; 4 School of Psychology, University of Surrey, Guildford GU2 7XH, UK; s.sabatini@surrey.ac.uk; 5 Applied Statistics Research Group, Department of Mathematics, Physics and Electrical Engineering, Northumbria University, Newcastle upon Tyne NE1 8ST, UK; eduwin.pakpahan@northumbria.ac.uk; 6 Dementia Centre of Excellence, Curtin enAble Institute, Curtin University, Perth, WA 6102, Australia; m.alshahrani12@postgrad.curtin.edu.au (M.A.); mario.siervo@curtin.edu.au (M.S.); 7 Non Communicable Diseases, Nutrition Research Division, icddr,b, Mohakhali, Dhaka 1000, Bangladesh; anaheed@icddrb.org; 8 School of Population Health, Curtin University, Perth, WA 6102, Australia

**Keywords:** dementia, risk factors, comorbidity

## Abstract

Dementia is a leading cause of disability and death globally. Individuals with diseases such as cardiovascular, cardiometabolic and cerebrovascular disease are often at increased dementia risk. However, while numerous models have been developed to predict dementia, they are often not tailored to disease-specific groups. Yet, different disease groups may have unique risk factor profiles and tailored models that account for these differences may have enhanced predictive accuracy. In this review, we synthesise findings from three previous systematic reviews on dementia risk model development and testing to present an overview of the literature on dementia risk prediction modelling in people with a history of disease. Nine studies met the inclusion criteria. Currently, disease-specific models have only been developed in people with a history of diabetes where demographic, disease-specific and comorbidity data were used. Some existing risk models, including CHA_2_DS_2_-VASc and CHADS_2_, have been externally validated for dementia outcomes in those with atrial fibrillation and heart failure. One study developed a dementia risk model for their whole population, which had similar predictive accuracy when applied in a sub-sample with stroke. This emphasises the importance of considering disease status in identifying key predictors for dementia and generating accurate prediction models for dementia.

## 1. Introduction

Risk prediction tools based on algorithms that aggregate risk factor profiles have been well integrated into routine clinical practice and many healthcare settings to inform decision making, with a key example being cardiovascular disease prevention [1]. To date, >50 different risk models for predicting incident dementia exist, typically incorporating demographic, health, lifestyle and genetic variables. Statistical methods used for developing risk models have evolved over time, including logistic/Cox regression, competing risk models (e.g., Fine–Gray method) and more recently, machine learning/artificial intelligence methods. However, current models developed within population-based settings are often characterised by varying predictive accuracy (c-statistic range: 0.49 to 0.96) and limited external validation [2,3,4,5]. Further, they are usually not tailored to disease-specific groups except for some conditions, including, for example, stroke and atrial fibrillation, with notable absences for other conditions such as Parkinson’s disease.

However, it is well established that people with diseases such as cardiovascular, cerebrovascular or metabolic diseases are at greater risk of dementia compared to disease-free individuals [6,7]. Although the mechanisms underlying these associations are not yet clear, they are complex and multifactorial, often driven by the specific condition, e.g., ischemic injury increasing the risk of dementia in people with stroke or insulin resistance increasing dementia risk in diabetics. Therefore, it may be that when developing dementia risk prediction models, it will be important to consider disease status and identify key predictors in different disease groups, rather than simply categorising individuals as disease positive vs. negative alongside other variables in a single model. Clinically, disease-specific risk models can help stratify patients to assessments and interventions particularly where their comorbidities (for example, stroke) increase their risk of subsequent dementia.

When models developed for predicting dementia risk in the general population have been externally validated in disease-specific cohorts, there have been examples of poor generalisability. For example, a study looked to externally validate population-based dementia models in a harmonised stroke cohort [8]. When the Cardiovascular Risk Factors, Aging and Dementia score, the Australian National University Alzheimer Disease Risk Index and the Brief Dementia Screening Indicator were mapped in stroke cohorts, the predictive accuracy for dementia was poor-to-low with a c-statistic values ranging from 0.53 to 0.66 across the three prediction models [8]. However, many of these models incorporate risk factors to predict dementia in the general population, such as physical inactivity or vascular risk factors. But those with comorbidities such as stroke or pre-existing cardiovascular disease will already have a number of these risk factors. This means that such variables may not be as predictive in these disease-specific populations compared to the disease-free population. It may be that models need to be developed in disease-specific cohorts and with disease-specific variables in the model itself to improve predictive accuracy.

Therefore, in this review, we synthesise findings from our previous systematic reviews [2,3,4] on dementia risk prediction models to provide an overview of model development and testing in disease-specific groups. The aim is to determine whether there is a possible need for developing disease-specific models to ensure accurate and reliable dementia prediction.

## 2. Materials and Methods

In this study, we synthesise findings from our three systematic reviews on dementia risk prediction model development and testing focusing specifically on those models developed or tested in a disease-specific group. A follow-up search was also conducted capturing all the literature up to November 2024 to identify any additional systematic reviews on dementia risk prediction in disease-specific groups that were missed in the previous search.

In brief, in each review, searches were conducted in electronic databases (e.g., EMBASE, Medline, Scopus and Web of Science) covering all the literature from the inception of each database until 2022. In general, the following terms were included in the search strategy across the reviews and mapped to Medical Subject Headings (MeSHs) where appropriate: dementia, Alzheimer disease, predict, develop, incident, sensitivity, specificity, ROC, area under the curve (AUC) and concordance statistic. Articles were included if (1) the sample was population-based; (2) the study examined the risk of incident late-life dementia (i.e., dementia at age ≥ 65 years); and (3) model evaluation metrics were reported, e.g., area under the curve [AUC] or c-statistic. In this review, we also only selected articles where a model was developed or tested in a group stratified by disease status (i.e., disease-positive vs. disease-negative). There was no restriction on the disease type or dementia outcome. Data were extracted independently by two authors (ET and BCMS), including sample size, study location, follow-up interval, disease state investigated, dementia outcome, model candidate predictor variables and model performance indices (e.g., c-statistic/AUC, calibration and external validation results). Like our reviews, models were classified as being poor (c-statistic < 0.60), low (c-statistic range: 0.60 to 0.69), moderate (c-statistic range: 0.70–0.79), good (c-statistic range: 0.80–0.89) and excellent (c-statistic ≥ 0.90). We also assessed the risk of bias of included articles using a modified version of the Newcastle–Ottawa Scale for non-randomised studies [9]. The modified version excluded items that describe non-intervention cohorts. The studies were assessed based on selection, comparability, and outcome criteria and could achieve a maximum of six stars (compared to the original nine).

## 3. Results

Across the three reviews, 120 articles were included. From these, seven studies met the inclusion criteria. Two further studies (citing three models) were found in an updated search, giving a total of nine included studies for this review, but no additional systematic reviews on the topic were identified. The key characteristics of each study including disease-group, predictor variables included in each model, follow-up time and model performance indices are shown in Table 1.

In summary, only four disease areas have looked at dementia risk, which include diabetes, stroke, heart failure and atrial fibrillation. Diabetes was the only condition where new models were developed in a disease-specific cohort (n = 4). Here, the models generally performed well even when validated in another population. However, a current limitation in this field is that most of these disease-specific models in other comorbidities have tended to validate existing non-dementia models to predict dementia. A common example includes the CHA_2_DS_2_-VASc score, which was originally developed to predict stroke risk in the context of atrial fibrillation.

### 3.1. Diabetes

Diabetes is the only condition where tailored dementia prediction models have been developed (n = 4 models [10,11,12,13]). One model [10] incorporated diabetes-specific variables (microvascular disease and diabetic foot), disease comorbidities (cerebrovascular disease, cardiovascular disease, acute metabolic events, and depression) and demographic information (age and education) for predicting incident dementia over 10 years in diabetic patients in the USA. The model had good discriminative accuracy (c-statistic = 0.74), good external validity (c-statistic = 0.75) and was well calibrated (Hosmer-Lemeshow x^2^ = 15.1). Another model was developed for predicting incident dementia at 10-year follow-up in Chinese type 2 diabetics that incorporated demographic factors (age and sex), diabetes-related variables (duration of type 2 diabetes in years, body mass index and variation in fasting plasma glucose, variation in HbA1c, and anti-diabetes medications use including oral only, insulin or insulin oral agent), and disease comorbidities (stroke, hypoglycaemia, postural hypertension, and coronary artery disease) [11]. The model’s discriminative accuracy for predicting dementia at 3-year (AUC = 0.82; 95% CI: 0.80–0.84), 5-year (AUC = 0.79; 95% CI: 0.77–0.81) and 10-year (AUC = 0.76; 95% CI: 0.75–0.77) follow-up in the derivation sample was moderate-to-good. When internally validated, the model showed good transportability across all three time points (3-year AUC = 0.84 (95% CI: 0.80–0.88), 5-year AUC = 0.80 (95% CI: 0.78–0.83) and 10-year AUC = 0.75 (95% CI: 0.73–0.77)) and was well calibrated.

Mehta et al. [12] developed the RxDx-Dementia risk index for older aged type 2 diabetics with hypertension using a primary care dataset in the UK. The model incorporated demographics (age and sex) and disease comorbidities (myocardial infarction, congestive heart failure, coronary and peripheral vascular disease, cerebrovascular disease, chronic pulmonary disease, rheumatologic disease, peptic ulcer disease, renal disease/end stage renal disease, mild liver disease/moderate or severe liver disease, any malignancy, including lymphoma and leukaemia/metastatic solid tumour, epilepsy, hyperlipidaemia, Parkinson’s disease, cardiac disease ASCVD (atherosclerotic cardiovascular disease), glaucoma, transplantation, thyroid disorder, gout, Crohn’s and ulcerative disease, pain and inflammation/pain, depression, psychotic illness, bipolar disorders, anxiety, and tension). The model performed well in the development (c-statistic = 0.81; 95% CI: 0.80–0.81) and internal validation (c-statistic = 0.81; 95% CI: 0.80–0.81) datasets. When tested in individuals with diabetes but no history of hypertension, the model showed even better discriminative ability (c-statistic = 0.86; 95% CI: 0.84–0.87). However, this model did not outperform a model consisting of age and gender only (c-statistic derivation sample = 0.78; 95% CI 0.77–0.79 vs. c-statistic validation sample = 0.83; 95% CI: 0.81–0.84). In contrast, the RxDx score did outperform the Charlson comorbidity score (c-statistic = 0.78; 95% CI: 0.78–0.79) and the chronic disease score (c-statistic = 0.79; 95% CI: 0.78–0.80).

The most recent model was developed for predicting incident dementia and vascular dementia in type 2 diabetics in Hong Kong [13]. This incorporated demographics (age), blood tests (total cholesterol (mmol/L, fasting blood glucose), medication use (calcium channel blocker use, diuretics, antiplatelet use) and medical history (ischemic stroke and coronary heart disease). The AUC for this model was lower than the other diabetes-specific models for incident all-cause dementia (AUC = 0.69). The AUC to predict vascular dementia was also 0.69.

### 3.2. Stroke

In one study, a model was developed in the general population and then assessed specifically in stroke patients [14]. The development sample included n = 267,855 participants, where 2.4% (n = 6516) progressed to dementia at 8-year follow-up. This study used a validated approach to estimate cognitive symptoms in narrative notes and adjusted for age, sex, race (Caucasian) and the Charlson comorbidity index [14]. The discriminative accuracy of the model was low (c-statistic = 0.61; 95% CI: 0.60–0.61). External validation of the model in a second academic medical centre reported a c-index of 0.65 (95% CI: 0.64–0.66). When both the development and validation cohorts were pooled, the c-index was 0.62. In individuals who had a primary discharge diagnosis of stroke (n = 18,681), the discriminative accuracy of the model was like that reported in the development and validation samples (c-statistic stroke sample = 0.59; 95% CI: 0.57–0.61).

### 3.3. Atrial Fibrillation (AF)

Two studies [15,16] explored the feasibility of repurposing the CHA_2_DS_2_-VASc model [19], which was originally designed to assess the risk of stroke in patients with AF and guide anticoagulant therapy decisions for the prediction of incident dementia. The CHA_2_DS_2_-VASc model incorporates age, history of hypertension, diabetes, recent cardiac failure, vascular disease (myocardial infarction or peripheral artery disease), sex, history of a stroke and transient ischemic attack (TIA). Importantly, all variables have been included in previous dementia prediction models developed for use in the whole population [2,3]—what is unique is the scoring system. In one study, the accuracy of the CHA_2_DS_2_-VASc model for predicting incident dementia in AF patients (N = 332,665; n = 29,012 incident dementia cases over 15-year follow-up) was low (c-statistic = 0.61; 95% CI: 0.61–0.61) [15]. In the second study [16] (n = 74,081; n = 449 dementia cases), the CHA_2_DS_2_-VASc risk model was stratified by (i) sex and (ii) Intermountain Risk Score (IMRS), which uses complete blood count and a basic metabolic profile for predicting mortality. The CHA_2_DS_2_-VASc had better accuracy for predicting dementia in women (c-statistic = 0.73; 95% CI: 0.64–0.81) compared to men (c-statistic = 0.65; 95% CI: 0.54–0.77) over 10-year follow-up. The higher accuracy in women is perhaps not surprising given that being female is associated with a higher risk of incident stroke in the context of AF according to the original risk score. When stratified by IMRS, the model performed better in females in the moderate IMRS group (c-statistic = 0.77; 95% CI: 0.66–0.87) compared to males in the moderate IMRS group (c-statistic = 0.67; 95% CI: 0.51–0.83). However, discriminative accuracy fell sharply when the CHA_2_DS_2_-VASc score was used to predict incident dementia in both women (c-statistic = 0.59; 95% CI: 0.41–0.76) and men (c-statistic = 0.27; 95% CI: 0.16–0.39) with a high IMRS [16]. When the IMRS alone was assessed to predict incident dementia, the discriminative accuracy was similar in men (c-statistic = 0.68; 95% CI: 0.59–0.78) and women (c-statistic = 0.69; 95% CI: 0.60–0.79) with minimal differences seen when stratified by CHA_2_DS_2_-VASc score. Liao et al. [15] also externally validated the CHADS_2_ score that incorporates age (≥75 years), hypertension, diabetes mellitus, heart failure and previous stroke or TIA. Like the CHA_2_DS_2_-VASc score, the CHADS_2_ was developed to predict stroke risk in people with AF [20]. The discriminative performance was poor and worse than for the CHA_2_DS_2_-VASc score (c-statistic = 0.59; 95% CI: 0.59–0.59) [15]. A final study validated the original Framingham Heart Study Dementia Risk Score (FDRS) and an extended FDRS model for predicting incident dementia in an AF cohort. The original FDRS model includes demographics (age, marital status), health status (body mass index) and medical history (prior stroke/TIA, prior diabetes, prior cancer). The extended model included the FDRS model variables plus hypertension, coronary artery disease, arrhythmia, heart failure, hyperlipidaemia and ejection fraction. Both the FDRS (c-statistic 0.74; 95% CI 0.72–0.76) and extended model (0.76; 95% CI: 0.74–0.78) showed moderate accuracy. Both models were also well calibrated [17].

### 3.4. Heart Failure

In one study, two models have been externally validated for predicting incident dementia in patients with heart failure (n = 387,595), including the CHA2DS2-VASc and the AHEAD score (incorporating AF, haemoglobin, age > 70 years, abnormal renal parameters, diabetes mellitus) [18]. The AHEAD score was originally validated for the estimation of the short- and long-term prognosis of patients hospitalised with acute heart failure [21]. The predictive accuracy for incident dementia over a mean follow-up of 2.91 years was poor for both the CHA_2_DS_2_-VASc (AUC = 0.61; 95% CI: 0.60–0.61) and the AHEAD (AUC = 0.55; 95% CI: 0.54–0.55) scores. In a further study, the FDRS score was applied in small sample of heart failure patients for predicting incident dementia over a mean of 3.5years of follow-up. The discriminative accuracy, however, was low (c-statistic 0.69; 95% CI: 0.66–0.72). When the original FDRS score was extended by including additional comorbidity variables, the discriminative accuracy was moderate (c-statistic = 0.72; 95% CI: 0.69–0.74) [17].

## 4. Discussion

As highlighted in this review, very few studies have focused on creating dementia risk models specifically for high-risk groups defined by the presence of disease, with only four conditions considered, including diabetes, stroke, AF and heart failure. To date, the only condition where models have been created specifically in a disease group is diabetes (n = 4). Generally, these models were found to have low to moderate predictive accuracy, although some models had c-statistic values > 0.80 (but none exceeded 0.90). Overall, the diabetes-specific models tend to outperform most dementia risk prediction models developed for use in the general population [2,3,4]. Where models developed for predicting non-dementia outcomes (such as stroke mortality) have been applied in disease-specific groups, such as people with AF and heart failure, the results show poor transportability for dementia outcomes (CHA_2_DS_2_-VASc: c-statistics range 0.59 to 0.61), unless they have been stratified by gender, where the accuracy does improve (CHA_2_DS_2_-VASc: c-statistics: 0.65 (men) and 0.73 (women)). In the only study that considered individuals with a history of stroke, it was found that a model developed for the general population performed similarly in the stroke vs. total population; however, the model’s accuracy was poor.

Regarding diabetes, two [10,11] of the four models combined disease-related factors (e.g., diabetic foot, HbA1c and anti-diabetes medication use) with health and demographic data and had moderate to good predictive accuracy for dementia (AUC/c-statistic range: 0.74–0.82) [10,11]). A further model [12] combined demographic variables with 31 disease conditions (including, for example, Parkinson’s disease) (see notes under Table 1) and had good predictive accuracy (c-statistic = 0.81). In contrast, one model that included fasting blood glucose in addition to non-diabetes specific health factors (i.e., total cholesterol, calcium channel blocker use, diuretics, antiplatelet use, ischemic stroke and coronary heart disease) had low accuracy (AUC = 0.69) but could distinguish risk by subtype (all-cause versus vascular dementia) [13]. As such, the results suggest that combining broad as well as diabetes-specific disease variables (e.g., microvascular disease, diabetic foot, fasting blood glucose, HbA1c) into prediction models leads to higher discriminative accuracy in this population. What is driving this result is not clear, but it could be that the combination of variables is capturing the multifaceted relationship between diabetes and dementia, thereby enabling more accurate predictions. Raised blood glucose levels (hyperglycaemia), even amongst those who are either non- or pre-diabetic, have been associated with cognitive impairment, particularly in areas such as verbal immediate and delayed memory [22]. Although mechanisms for this are unclear, it seems likely that glucose transfer across the blood–brain barrier causes damage to the brain, with areas such as the hippocampus being more sensitive to these detrimental effects [22].

One study did look to externally validate the FDRS model, which was originally developed to predict dementia but then validated in heart failure (c-statistic 0.69) and atrial fibrillation cohorts (c-statistic 0.74). Helpfully, discrimination levels increased when additional comorbidity data were added to the model in both the heart failure (c-statistic 0.72) and atrial fibrillation (c-statistic 0.76) cohorts [17].

Whether for other conditions, such as stroke and cardiovascular diseases, the inclusion of disease-specific variables in risk models enhances their accuracy remains to be tested. Indeed, in cerebrovascular disease, there are known characteristics of the stroke itself which can be predictive of dementia, such as stroke severity [23] and location [24]. For example, left-hemisphere strokes increase an individual’s risk of subsequent dementia [25]. For most individuals, the left hemisphere is dominant for language. It is an essential cognitive domain for completing neuropsychological or cognitive tests, and this may mean deficits are more obvious on cognitive testing [23]. Further, having multiple or recurrent stroke also increases one’s risk of cognitive decline post-stroke [25,26]. Besides the location, severity and number of strokes having a direct detrimental effect on brain parenchyma, evidence also suggests that pre-existing brain changes, such as small vessel disease, are also major contributors to cognitive decline post-stroke [23]. In cardiovascular disease, there is evidence that coronary heart disease, heart failure, AF, hyperlipidaemia and arterial stiffness [7] can increase one’s susceptibility to dementia and, if incorporated into a risk model, could possibly enhance predictive accuracy. Although disease-specific markers could increase model accuracy, this should be weighed up against their availability, cost and accessibility. This is particularly important if these models were to be used in resource-poor settings or in low- and middle-income countries.

Where models developed for other outcomes have been utilised for dementia prediction in AF and heart failure patients, model performance was poor. These scores predominately capture variables related to poor cardio-metabolic health, supporting the literature showing these are critical components in dementia risk [6]. However, while they appear to offer some predictive value, they fail to capture the key drivers of dementia risk in AF and heart failure patients. As highlighted above, to enhance dementia prediction in these groups, better characterisation of the disease-specific drivers underlying risk will likely be needed. It should be noted that if the original models were developed to predict dementia and then extended with additional variables, there was some improvement in the performance of these models, as highlighted by Manemann and colleagues [17].

### 4.1. Future Directions

Future research should look to develop and validate new, more accurate models in disease-specific populations, especially where disease-specific risk variables are likely to produce more accurate predictions of dementia risk. Existing dementia risk prediction models developed for the whole population could also possibly be extended through inclusion of disease-specific variables. Indeed, very few models had c-statistic values > 0.75, and to enable recommendations of clinical use, model predictive accuracy must be improved; model development stratified by disease status may be one approach to achieve this given the overall high performance of the diabetes-specific models.

Although this review focusses on four conditions commonly linked to dementia, there are many other conditions/diseases that increase one’s risk, such as Parkinson’s disease [27]. Therefore, new model development should consider a broader spectrum of conditions. It will also be helpful for future studies to utilise more advanced analytical approaches (e.g., machine learning/AI algorithms) that may be more successful in capturing complicated non-linear relationships between various risk factors, disease status and dementia onset. Such models could lead to improved risk stratification and enable earlier access to treatment.

A key consideration in creating new dementia risk prediction models for disease-specific populations is multimorbidity. For example, individuals with cardiovascular disease may be more likely to have diabetes and stroke. This raises the following questions: (1) In patients who have diabetes, stroke and cardiovascular disease, for example, how does one decide which disease-specific model to apply? (2) Are separate or combined models more accurate? Answering these questions will require an in-depth evaluation of the key component predictors for dementia in different disease groups (including their interaction to capture synergistic effects of comorbid conditions) to determine where overlap in risk factors might exist. Where overlap exists, a possible strategy might be to create base models for each condition (e.g., for stroke, the base model might include variables like stroke severity, location and number), which can then be combined with interaction terms to account for comorbidities. Achieving this, however, will require access to longitudinal data with well-characterised patient/disease populations across different world settings. Finally, a key aspect of implementing these models will be to consider the views of key stakeholders. Dementia risk discussions between patients and clinicians are likely to be complex and care is needed in how this information is communicated in the context of pre-existing comorbid conditions [28]. Resources and strategies need to be co-developed with patients to ensure the appropriate translation of these models. This is particularly important in conditions such as stroke where the patients (and their families) will need to adapt to their post-stroke selves. Discussing the risk of further decline in, for example, cognition needs to be handled sensitively and at the right time for the patient. Appropriate clinical pathways are also needed to know where in the pathway these models can be best utilised.

### 4.2. Strengths and Limitations

There are several key strengths of this review. First, the same systematic approach has been used across the review to ensure that all the relevant literature was captured over time. We also conducted an updated search to pick up additional studies beyond the published work. Second, we did not exclude by language, thereby reducing any bias associated with only presenting papers written in English. Most of the studies scored six stars (range 4–6) in a risk of bias assessment, which would indicate that the studies were of moderate or high methodological quality, with minimal bias observed in the reporting of the studies. There are also limitations. Given the large differences in the methodology used across studies and as few studies applied the same model, we were not able to perform a meta-analysis and therefore only a narrative synthesis (themed by disease groups) was possible. We only included population-based studies and therefore models developed in clinical samples are not represented here. Clinical studies are more likely to have access to more disease-specific variables and thus might lead to more accurate models. The limitation in using cohort studies would be that we would be limited to the inclusion/exclusion criteria set by the cohort and would therefore be less generalizable to that disease group—for example, if only minor stroke patients were included. Finally, new model development has only been undertaken in people with diabetes as the other studies externally validate previously developed models in disease-specific groups. While the results suggest that incorporating disease specific variables tends to lead to higher accuracy, whether this is true for all conditions remains to be established. Further model development in other disease areas is urgently needed to clarify this before implementation.

## 5. Conclusions

There is a significant gap in the development and testing of dementia risk prediction models in people with poor health. To accurately predict dementia risk in disease-positive groups, our results suggest that models that integrate unique disease-related factors that contribute to dementia risk may lead to more accurate predictions. These variables are likely to be different depending on the underlying disease. Due to the overlapping nature of some of these diseases, it may well be that future disease-specific models should focus on those at the greatest risk, such as stroke. Being able to accurately predict risk of dementia would enable healthcare providers to offer personalised, proactive and patient-centric care, making a significant impact on the burden of disease associated with this condition.

## Figures and Tables

**Table 1:** Characteristics of included studies.

Publication	Country	Baseline Sample Followed Up (Incident Cases)	Follow-Up (Years)	Predictor Variables Include in the Model	Range Development AUC/C-Statistic Range (95% CI) for Models Incorporating One or More of the Predictor Variables	Range Validation C-Statistic/AUC ^a^	Calibration	Main Analytical Method	Risk of Bias
DIABETES									
Exalto (2013) [10]	USA	29,961 type 2 diabetics (5173)	10	Age, education, microvascular disease, diabetic foot, cerebrovascular disease, cardiovascular disease, acute metabolic event, and depression	0.74	0.75	Yes, H-L x^2^ = 15.1	Cox proportional hazard model	******
Li (2018) (European journal) [11]	Taiwan	27,540 Chinese type 2 diabetics including n = 18,360 derivation dataset (853 dementia cases) and n = 1228 in the validation dataset (375 dementia cases)	Mean = 8.1	Age, sex, diabetes duration, BMI, variation (%) fasting plasma glucose, variation (%) HBa1c, stroke, hypoglycaemia, postural hypertension, coronary artery disease, and anti-diabetes medications	0.76 (0.75–0.77) to 0.82 (0.80–0.84)	0.75 (0.73–0.77) to 0.84 (0.80–0.88)	H-L all *p* > 0.05 (Excellent fit)	Cox regression	******
Mehta (2016) [12]	UK	Participants with diabetes and hypertension: 133,176 Participants with diabetes and no hypertension: 16,677 (validation sample)	9	Age, gender, RxDx-Dementia risk index ^a^, Charlson comorbidity score ^b^, chronic disease score ^c^	Age+Gender 0.78 (0.78–0.79) RxDx-Dementia Risk Index 0.81 (0.80–0.81) Charlson comorbidity score 0.78 (0.78–0.79) Chronic Disease Score 0.79 (0.78–0.80)	Age+Gender 0.83 (0.81–0.84) RxDx-Dementia Risk Index 0.86 (0.84–0.87) Charlson comorbidity score 0.83 (0.82–0.84) Chronic Disease Score 0.83 (0.82–0.85)	RxDx-Dementia risk index H-L test < 0.001 (Poor fit)	Cox regression	******
Chau (2023) [13]	Hong Kong	Type 2 diabetics	Median = 11.6	Age, total cholesterol (mmol/L), calcium channel blocker use, diuretics, antiplatelet use, Ischemic stroke, coronary heart disease, and fasting blood glucose	0.69 to 0.69	NA	NR	Multivariate Cox regression	******
STROKE									
McCoy (2020) [14]	USA	Total: 267,855 (6516) Stroke: 18,681 (NR)	8	Age, sex, white race, Charlson comorbidity index, cognitive symptom burden score (at discharge from narrative hospital notes)	0.59 (0.57–0.61)	NA	NR	Fine–Gray sub-distribution hazards model	******
ATRIAL FIBRILLATION									
Liao (2015) [15]	Taiwan	Participants with atrial fibrillation: 332,665 (29,012)	15	CHA2DS2-VASc ^d^ and CHADS2 ^e^	NA	CHA2DS2-VASc score 0.61 (0.61–0.61) CHADS2 score 0.59 (0.59–0.59)	NR	Cox regression	*****
Graves (2019) [16]	USA	74,081 (449) with atrial fibrillation	10	IMRS (complete blood count and basic metabolic panel) CHA2DS2-VASc score (as above) ^d^	NR	IMRS Women Overall: 0.69 (0.60–0.79) CHA2DS2-VASc < 1: Not enough events CHA2DS2-VASc 2: Not enough events CHA2DS2-VASc: 0.63 (0.53–0.74) Men Overall: 0.68 (0.59–0.78) CHA2DS2-VASc score < 1: Not enough events CHA2DS2-VASc 2: 0.68 (0.48–0.88) CHA2DS2-VASc 3: 0.57 (0.44–0.70) CHA2DS2-VASc Women Overall: 0.73 (0.64–0.82) Low IMRS: Not enough events Moderate IMRS: 0.77 (0.66–0.87) High IMRS: 0.59 (0.41–0.76) Men Overall: 0.65 (0.54–0.77) Low IMRS: Not enough events Moderate IMRS: 0.67 (0.51–0.83) High IMRS: 0.27 (0.16–0.39)	NR	Cox regression	******
Manemann (2023) [17]	USA	Atrial fibrillation patients: 4107 (736)	Mean = 3.7	Model 1: FDRS ^f^ Model 2: Model 1 + hypertension, coronary artery disease, arrhythmia, hyperlipidemia, arthritis, asthma, chronic kidney disease, chronic pulmonary disease, depression, osteoporosis, schizophrenia, and substance abuse disorder, sex, ejection fraction, smoking, education	NA	Model 1 0.74 (0.72–0.76) Model 2 0.76 (0.74–0.78)	Group-based measure of calibration indicated a mean standardised incidence ration of 0.994 (well-calibrated)	Cox proportional hazard model	******
HEART FAILURE									
Hu (2019) Comparison of CHA2 [18]	Taiwan	Heart failure patients: 387,595 (NR)	Mean = 2.91	CHA2DS2-VASc ^d^ AHEAD ^g^	CHA2DS2-VASc 0.61 (0.60–0.61) AHEAD 0.55 (0.54–0.55)	NA	NR	Cox proportional hazard model and Fine and Gray analysis (LASSO selection)	****
Manemann (2023) [17]	USA	Heart failure patients: 3052 (626)	Mean = 3.5	Model 1: FDRS ^f^ Model 2: Model 1 + hypertension, coronary artery disease, heart failure, hyperlipidemia, arthritis, asthma, chronic kidney disease, chronic pulmonary disease, depression, osteoporosis, schizophrenia, and substance abuse disorder, sex, smoking, and education	NA	Model 1 0.69 (0.66–0.72) Model 2 0.72 (0.69–0.74)	Group-based measure of calibration indicated a mean standardised incidence ration of 0.988 (well-calibrated)	Cox proportional hazard model	******

Acronym Key: AUC = area under the curve; BMI = Body Mass Index; FDRS = Framingham Heart Study Dementia Risk Score; HbA1c = Glycated Haemoglobin, H-L = Hosmer–Lemeshow; IMRS = Intermountain Risk Score; NA = not applicable; NR = not reported; TIA = transient ischemic attack; UK = United Kingdom; USA = United States of America; 95% CI = 95% Confidence Interval. Notes: ^a^ RxDx Dementia risk index incorporates myocardial infarction, congestive heart failure, coronary and peripheral vascular disease, cerebrovascular disease, chronic pulmonary disease, rheumatologic disease, peptic ulcer disease, renal disease/end stage renal disease, mild liver disease/moderate or severe liver disease, any malignancy, including lymphoma and leukaemia/metastatic solid tumour, epilepsy, hyperlipidaemia, Parkinson’s disease, cardiac disease ASCVD, glaucoma, transplantation, thyroid disorder, gout, Crohn’s and ulcerative disease, pain and inflammation/pain, depression, psychotic illness, bipolar disorders, anxiety, and tension; ^b^ Charlson comorbidity score incorporates 17 variables (age, myocardial infarction, congestive heart failure, peripheral vascular disease, cerebrovascular disease, dementia, chronic pulmonary disease, rheumatologic disease, peptic ulcer disease, liver disease, diabetes, hemiplegia or paraplegia, moderate to severe renal disease, malignancy, leukaemia, lymphoma, AIDS; ^c^ Chronic disease score incorporates the prescription-based comorbidity index where outpatient pharmacy dispensing data were used to identify specific disease conditions. The CDS contains 29 disease conditions and weights were assigned to each disease condition. ^d^ CHA_2_DS_2_-VASc incorporates age (65–74 years), history of hypertension, diabetes, recent cardiac failure, vascular disease (myocardial infarction or peripheral artery disease), female gender, history of stroke, transient ischemic attack, and age ≥ 75 years; ^e^ CHADS_2_ incorporates age ≥ 75 years, hypertension, diabetes mellitus, and heart failure, previous stroke, or transient ischemic attack; ^f^ FDRS incorporates age, marital status, BMI, prior stroke/ transient ischemic attack, prior diabetes, and prior cancer; ^g^ AHEAD incorporates 5 variables (*A*—atrial fibrillation; *H*—haemoglobin < 130 g/L for men and 120 g/L for women (anaemia); *E*—elderly (age > 70 years); *A*—abnormal renal parameters (creatinine > 130 μmol/L); D—diabetes mellitus) in a scoring system. Risk of bias assessment is based on selection, comparability, and outcome criteria (maximum of 6 *), using a modified version of the Newcastle–Ottawa Scale for non-randomised studies [9].

## Data Availability

All data contained within this article are available from our previously published systematic reviews.
